# Primary Cutaneous CD4 Small/Medium T-Cell Lymphoproliferative Disorder Following COVID-19 Vaccination—What Do We Know about Lymphoproliferative Disorders and Cutaneous Lymphomas after COVID-19 Vaccination? A Report of an Atypical Case and a Review of the Literature

**DOI:** 10.3390/life14030386

**Published:** 2024-03-14

**Authors:** Francisco Javier De la Torre-Gomar, Jose María Llamas-Molina, Maria Dolores Pegalajar-García, Carmen Pérez-Valencia, Alejandro Carrero-Castaño, Ricardo Ruiz-Villaverde

**Affiliations:** 1 Dermatology Department, San Cecilio University Hospital, 18016 Granada, Spain; josellamas94@gmail.com (J.M.L.-M.); md.pegalajar.g@gmail.com (M.D.P.-G.); ismenios2005@gmail.com (R.R.-V.); 2 Radiology Department, San Cecilio University Hospital, 18016 Granada, Spain; carmenpval17@gmail.com; 3 Pathology Department, San Cecilio University Hospital, 18016 Granada, Spain; acarrero28@hotmail.com

**Keywords:** CD4+ small/medium-sized T-cell lymphoproliferative disorder, COVID-19 vaccine, cutaneous lymphoproliferative disorder, cutaneous lymphoma

## Abstract

The association between Primary cutaneous CD4 small/medium T-cell lymphoproliferative disorder (PCSM-TCLPD) and COVID-19 immunization has been sparsely documented in the medical literature. Reviewing the literature, albeit infrequently, we can find cases of the recurrence and new onset of lymphoproliferative processes and cutaneous lymphomas following the COVID-19 vaccine. Many of the entities we encounter are classified as cutaneous lymphoproliferative disorders. The prevailing hypothesis suggests that the predominant cutaneous reactions to SARS-CoV-2 vaccines may stem from T-cell-mediated immune activation responses to vaccine components, notably messenger RNA (mRNA). Specifically, it is posited that the presence of cutaneous lymphoid infiltrates may be linked to immune system stimulation, supported by the absence, to date, of instances of primary cutaneous B-cell lymphoma following mRNA vaccination. Within this context, it is imperative to underscore that the etiological association between PCSM-TCLPD and COVID-19 vaccination should not discourage vaccination efforts. Instead, it underscores the necessity for continuous surveillance, in-depth investigation, and comprehensive follow-up studies to delineate the specific attributes and underlying mechanisms of such cutaneous manifestations post vaccination.

## 1. Introduction

SARS-CoV-2 vaccination remains one of the most effective measures to minimize the impact of the global pandemic. Since December 2020, COVID-19 vaccines have been available worldwide. The mRNA vaccines BNT162b2 (Comirnaty, Pfizer/BioNTech, New York, NY, USA), mRNA1273 (Moderna, Cambridge, MA, USA), and AZD1222 (AstraZeneca, Wilmington, DE, USA) were the first to receive authorization. These vaccines elicit both innate and acquired immune responses through the use of mRNA, viral vectors, and inactivated viruses [1]. Various skin reactions have been documented following COVID-19 immunization. Common skin reactions associated with vaccines include local reactions, urticaria, morbilliform rash, and erythromelalgia. The median time between the first dose and the onset of skin symptoms is 7 days, while the median time between the second dose and the appearance of skin reactions is only 1 day [2].

The pathogenesis of cutaneous reactions following mRNA COVID-19 vaccination can be categorized into four groups: Type I hypersensitivity (including urticaria, angioedema, and anaphylaxis), Type IV hypersensitivity (including delayed large local skin reactions known as “COVID arm”, dermal filler inflammatory reactions, morbilliform, and erythema multiforme-like rashes), autoimmune-mediated reactions (involving lupus erythematosus, bullous pemphigoid, leukocytoclastic vasculitis, vitiligo, and alopecia areata), or other etiologic mechanisms (encompassing entities such as pityriasis rosea, chilblain-like lesions, herpes zoster, or alopecia).

In addition to these manifestations, it is crucial to consider primary cutaneous lymphoproliferative disorders (PCLDs) that may be associated with the SARS-CoV-2 vaccine. However, many of their characteristics remain unknown due to their infrequency and limited study. The relationship between PCLDs and SARS-CoV-2 appears complex, and the exact pathogenic mechanisms, if any, are not fully understood. Furthermore, we must acknowledge that this relationship could also be coincidental.

Recent reports suggest spontaneous regression of cutaneous lymphomas after vaccination. Among the publications in this regard is the article by Gambichler et al., reporting marked regression of a primary cutaneous anaplastic large-cell lymphoma one week after receiving the first dose of the SARS-CoV-2 vaccine (BNT162b2) [3]. Aouali reported a complete remission of primary cutaneous follicular B-cell lymphoma after the administration of the first dose of the COVID-19 vaccine (AZD1222) [4].

In genetically predisposed individuals, these results suggest that the vaccine may enhance antitumor immunity. This response may be analogous to that induced by the BCG vaccine, particularly in the treatment of metastatic melanoma, as it activates the immune system and destroys tumor cells [3,4,5].

Conversely, this immune stimulus may act as a trigger for cutaneous lymphoproliferative processes, considering that lymphomagenesis is closely linked to the physiological immune functions of T cells in the skin. In this context, Brumfield et al. recently reported the recurrence of a primary cutaneous CD30-positive lymphoproliferative disorder two days after the first vaccination with the Pfizer-BioNTech COVID-19 vaccine in the ipsilateral arm [6]. Panou et al. published two cases of clinical reactivation in cutaneous T-cell lymphoma after the administration of the AZD1222 vaccine. One case involved the recurrence of type-A lymphomatoid papulosis (LyP) 10 days after administration of the first dose in a patient with a history of LyP and mycosis fungoides who had been in remission for 10 years. In the other case, a patient with folliculotropic mycosis fungoides with previously well-controlled disease progressed to CD30+ large cell tumor (LCT) stage MF after administration of the second dose [7].

Subsequently, reports of lymphoproliferative processes and cutaneous lymphomas following vaccination also emerged. To date, this remains an infrequent and poorly understood condition with limited clinical cases and a small number of case series in the available literature. In this context, we present a case of Primary cutaneous CD4 small/medium T-cell lymphoproliferative disorder (PCSM-TCLPD) following SARS-CoV-2 vaccination, and we conduct a review of the lymphoproliferative processes and primary cutaneous lymphomas following this potential trigger.

## 2. Materials and Methods

Our search spanned from October 2021 to February 2023. In addition to the literature review, we present a previously unreported case involving a Primary cutaneous CD4 small/medium T-cell lymphoproliferative disorder (PCSM-TCLPD) following COVID-19 vaccination.

## 3. Case Report and Results

**Figure 1:**
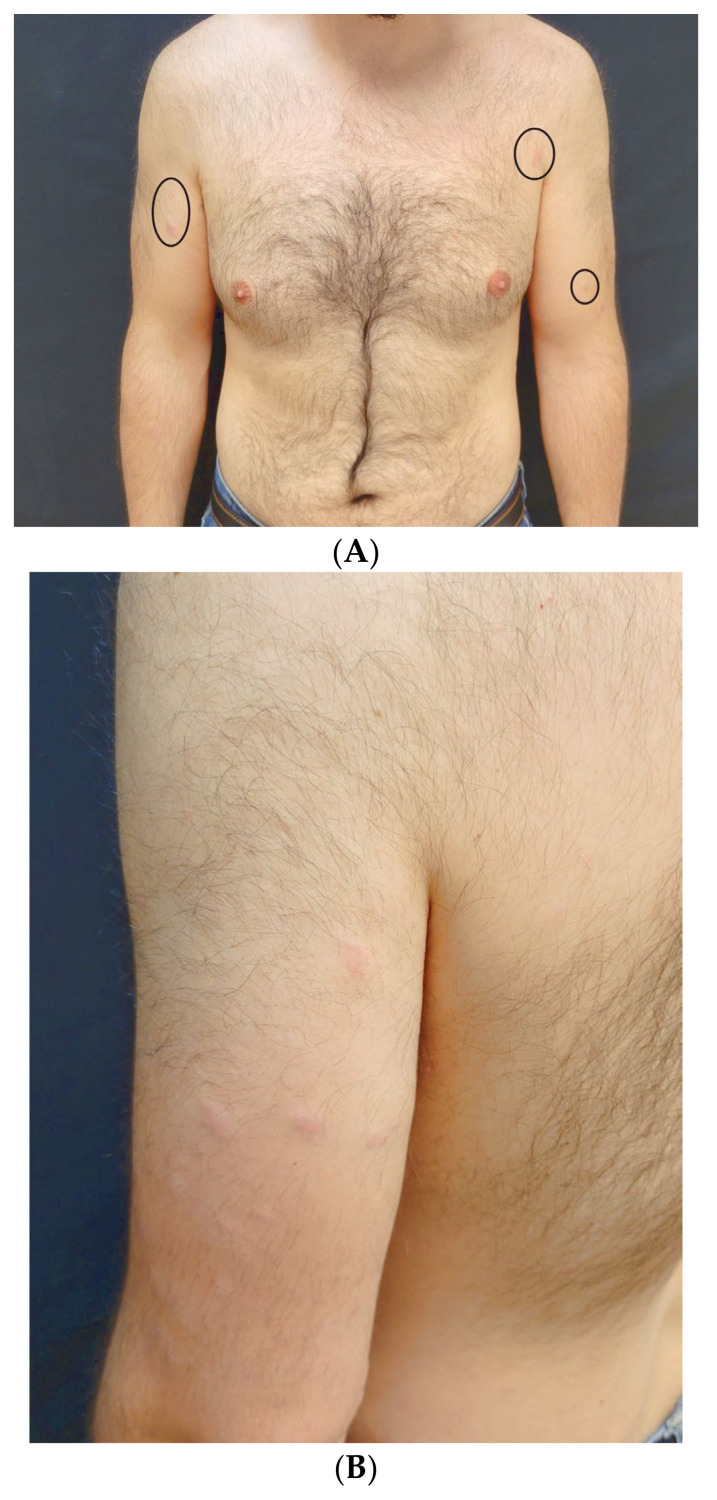

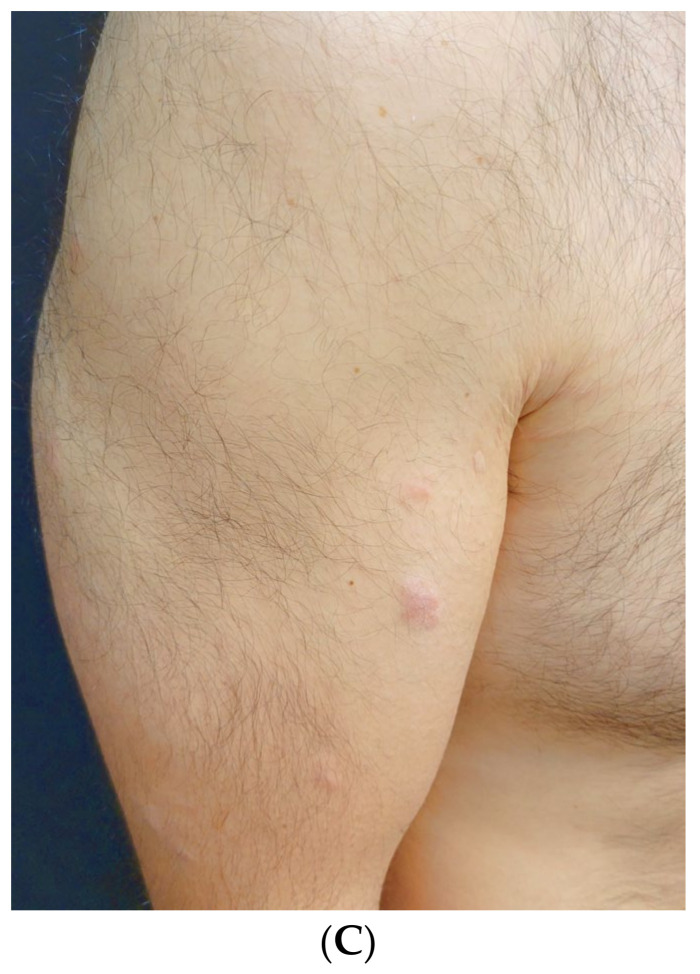
Rose-colored, erythematous, well-demarcated nodules distributed on chest—located inside the circles—(**A**), left arm (**B**), and right arm, where the histological study of the more medial lesion was conducted (**C**).

A 30-year-old man with no significant medical history presented to our Dermatology Unit with a 12-month history of cutaneous papular and nodular eruptions distributed over the left arm, right arm, right side of the chest, right dorsal back, and right lumbar back. The lesions appeared 7 days after receiving the first dose of the COVID-19 vaccine (BNT162b2^®^, Pfizer/BioNTech, New York, NY, USA). Three weeks later, he received the second dose of the same vaccine without developing new lesions. Physical examination revealed slightly painful, upon contact, and non-pruritic erythematous nodules symmetrically distributed in the mentioned locations (Figure 1A–C).

**Figure 2:**
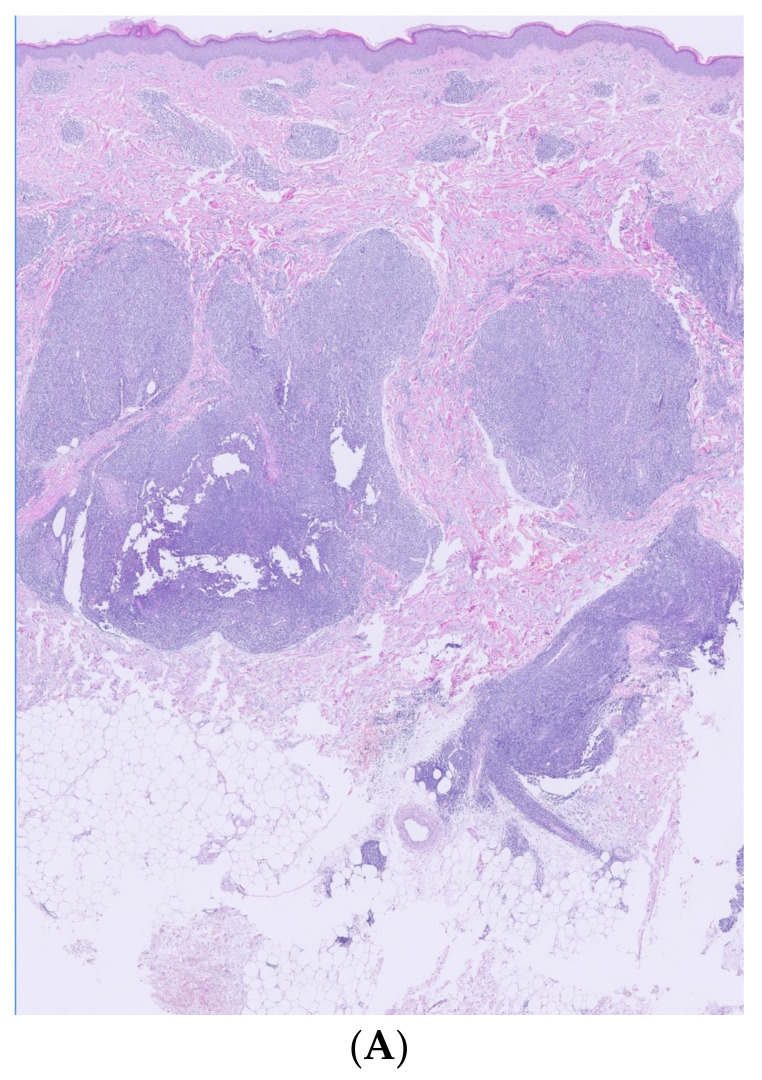

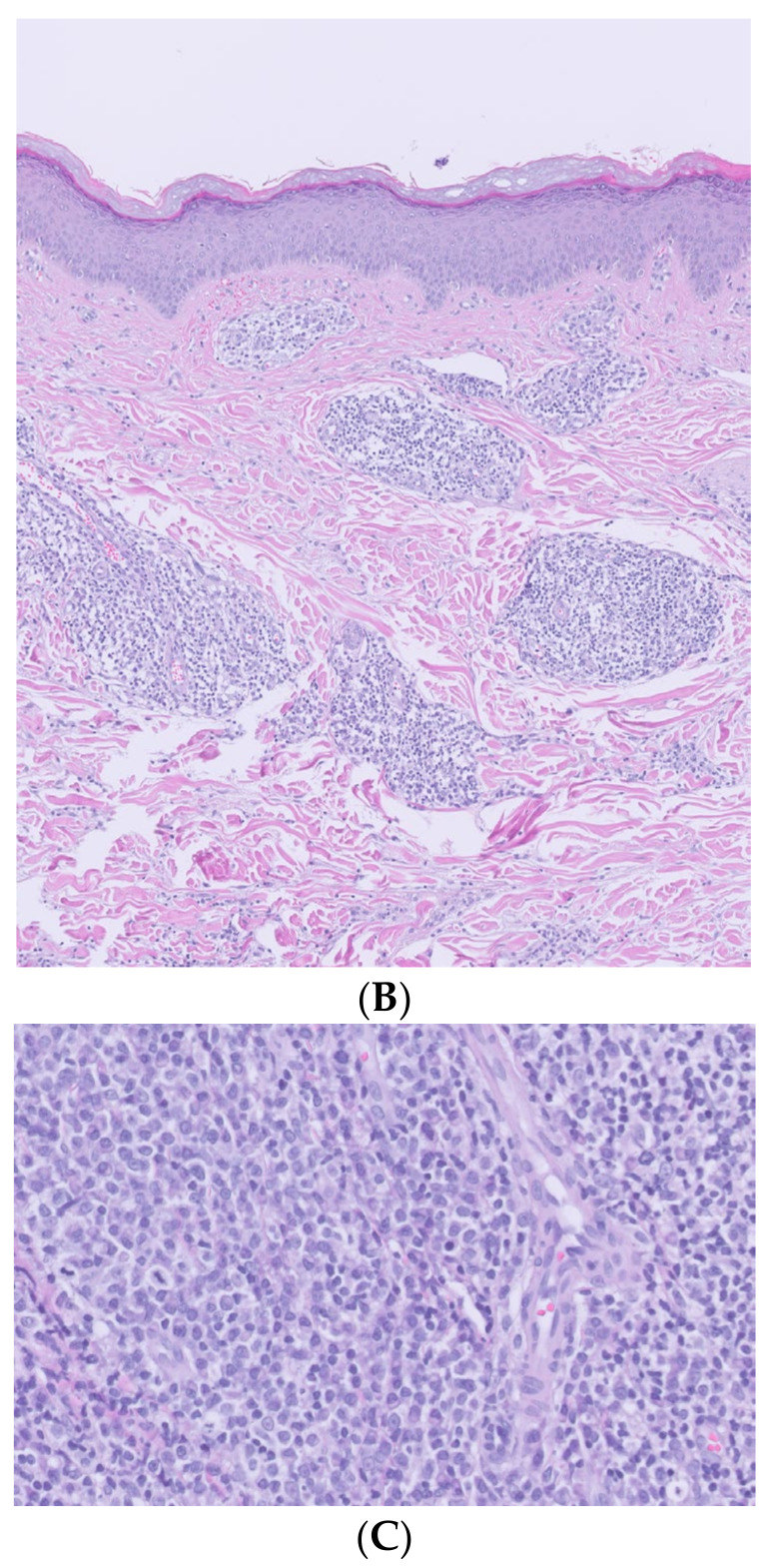
Histological examination. (**A**) (Hematoxylin/eosin ×2): large accumulations of lymphoid infiltrate, with perianexial and perivascular distribution located in superficial and deep dermis in minimal contact with hypodermis. (**B**) (Hematoxylin/eosin ×10): absence of epidermotropism, showing a respected Grenz zone. (**C**) (Hematoxylin/eosin ×40): dermal infiltrate composed of a heterogeneous mixture of mainly T and B lymphocytes.

**Figure 3:**
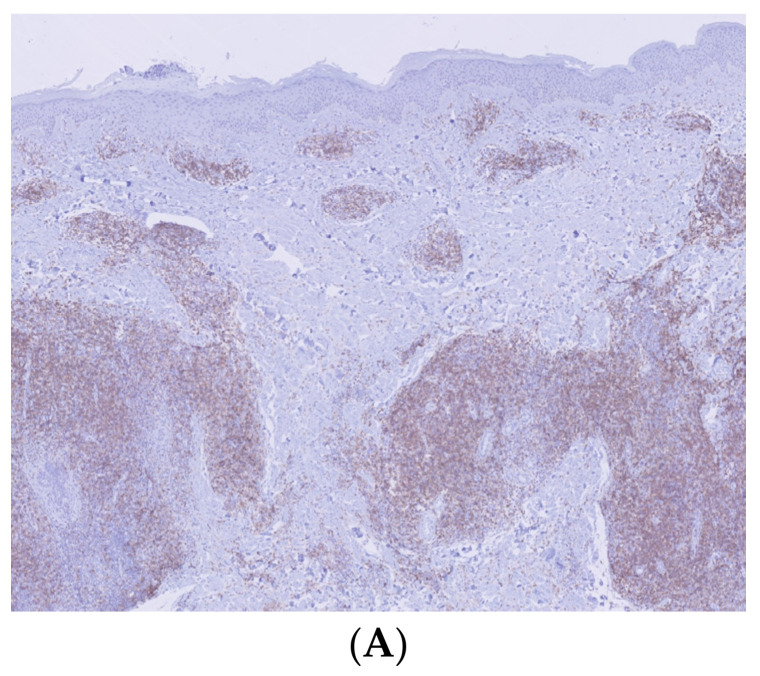

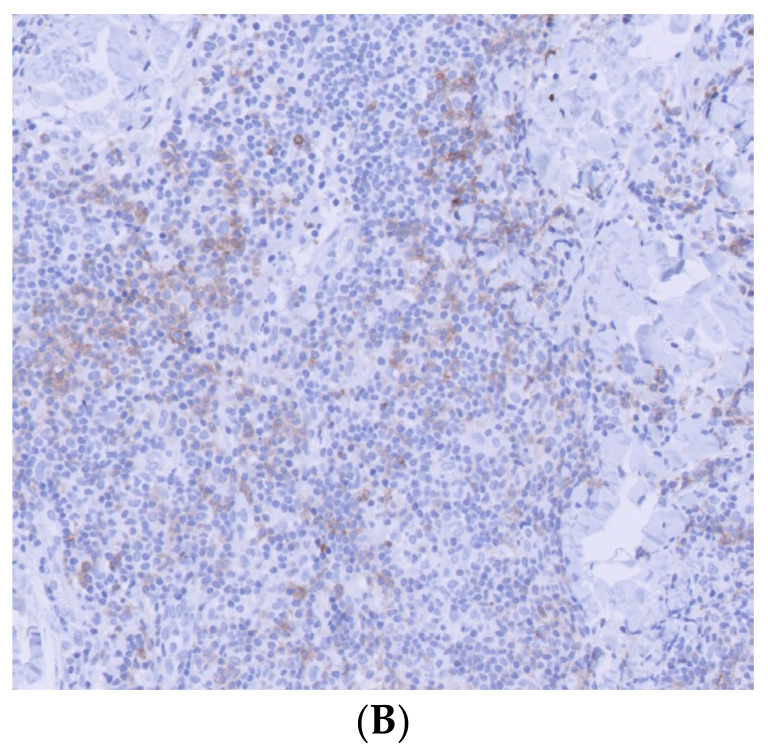
Immunohistochemistry. (**A**) (Immunohistochemistry ×2): positive CD4 lymphocyte infiltrate. (**B**) (Immunohistochemistry ×20): small/medium cells with moderate cytological atypia and intermediate-sized elements showing positivity for the PD1 marker.

Histological and immunohistochemical examination of a lesion located on the right arm revealed a dense confluent lymphoid infiltrate that was predominantly perivascular and periadnexal, distributed in the superficial and deep dermis. The infiltrate consisted of small/medium CD3+/CD4+/CD5+/CD7+ cells with moderate cytologic atypia. Intermediate-sized elements were positive for PD1. Molecular analysis showed a positive rearrangement for TCR gamma, with T-cell clonality present in the analyzed lymphoid population. The proliferation did not exhibit epidermotropism, leaving a respected Grenz zone (Figure 2 and Figure 3).

Based on the clinicopathologic findings and the temporal relationship, a diagnosis of PCSM-TCLPD after COVID-19 vaccination was established. Considering the atypical presentation with generalized skin lesions, staging imaging was deemed necessary, and a positron emission tomography/computed tomography (PET/CT) scan was performed, revealing no pathological findings. As the patient showed no evidence of systemic involvement, he was followed up and treated with intralesional triamcinolone acetonide infiltration at a concentration of 40 mg/mL in each lesion in a single administration. The lesions resolved two months after infiltration. Fourteen months later, the patient has not reported any other symptoms.

PCSM-TCLPD is a rare subtype of primary cutaneous peripheral T-cell lymphoma recognized as a provisional entity included in the 2005 WHO-EORTC classification and its 2018 update. The association between PCSM-TCLPD and COVID-19 vaccination has been extremely rare in the literature [5]. Additionally, cases of new-onset or relapse of other lymphomas or lymphoproliferative disorders have been reported in recent years, as summarized in Table 1 [5,6,7,8,9,10,11]. Notably, these cases involve 15 males and 9 females, with a median age of 60 years (ranging from 20 to 80 years). Interestingly, in 20 out of 24 reported patients, skin lesions occurred after BNT162b2 vaccination, mostly after the second dose. The median time from the first vaccine dose to the onset of BNT162b2 was 17.5 days. Reported cases after ChAdOx1 nCoV-19 vaccination include two relapses, one of CD30LCT-MF and another of LyP type-A, along with a new onset of LyP type-D. Both the mRNA-1273 and BNT162b2 mRNA vaccines have demonstrated similar efficacy and immunogenicity [9]. However, it is noteworthy that only one case of new-onset primary cutaneous γ/δ T-cell lymphoma has been reported with mRNA-1273. When compared to other cases in the literature, this instance, along with PCCD30-LPD, CD30LCT-MF, SS, and erythrodermic MF, represents the aggressive entities described. Analyzing the cases outlined above, relapses were documented in PCCD30-LPD (one case), CD30LCT-MF (two cases), LyP type-A (two cases), PCSM-TCLPD (one case), SS (one case), and erythrodermic MF (one case). Conversely, new cases were described in entities such as LyP type-A (three cases), CD30LCT-MF (one case), CLH (one case), PCSM-TCLPD (two cases), SS (one case), and PCGDTCL (one case). The manifestations described are highly variable, often transient, tend to resolve spontaneously, and can be successfully treated with standard therapies when indicated. Similarly, most of the described entities are considered cutaneous lymphoproliferative disorders (17/24). These data support the notion that the immunostimulatory effect induced by vaccination may be more associated with the induction of indolent/intermediate entities. The median time between the last vaccine dose and lesion development was 15 days (ranging from 2 to 42). For new-onset entities, the median time was 19.2 days, while for relapses, it was 12.56 days.

## 4. Discussion

In the recent WHO classification (2016), PCSM-TCLPD was proposed to designate an entity whose etiopathogenesis and prognosis are currently under discussion [12]. It is an infrequent, indolent, and relatively poorly characterized disease, lacking clear diagnostic or treatment guidelines. The classic clinical manifestation of PCSM-TCLPD is an asymptomatic single papule or nodule, mainly located on the head and neck or upper trunk, typically affecting middle-aged or elderly patients. Although uncommon, locally multifocal presentations (as in our patient) have been reported in approximately 7% of patients. Involvement of the lower extremities, occurrence in younger patients, and the presence of associated symptoms such as pruritus have also been very rarely described [12,13]. The main differential diagnoses for this condition include basal cell carcinoma, adnexal tumors and cysts, and Jessner’s lymphocytic infiltration, owing to its small size and tendency to occur on the head and neck [13].

Histopathology usually reveals a dermal infiltrate that is diffuse or nodular and not epidermotropic. The infiltrate consists of mildly atypical lymphocytes that are CD4+ and CD3+. These lymphocytes can form clusters or rosettes and express PD-1+, CXCL13, or BCL-6. The Ki-67 index should be lower than 20%. Additionally, examining the T-cell receptor (TCR) for monoclonal rearrangements in β- and γ-chains can be valuable in establishing a diagnosis [13]. Clinical management of PCSM-TCLPD is variable. Imaging studies and bone marrow examinations have limited diagnostic value and may not be necessary in all patients [14]. In atypical cases, a complete blood count with differentiation, flow cytometry, and imaging of the chest, abdomen, and pelvis may be considered. The imaging workup involves PET/CT scans, where the most commonly reported abnormality is low or nonspecific fluorodeoxyglucose enhancement. In one of the largest published reviews, abnormal uptake was found in 30.4% of PET/CT scans, but none of these findings was considered clinically significant or affected patient management [14]. While bone marrow biopsy is commonly used in the staging of hematologic malignancies, it is not necessary in this entity [12]. Solitary forms of PCSM-TCLPD have an indolent clinical course, with most patients presenting with localized disease.

PCSM-TCLPD should be distinguished from both B-cell and T-cell pseudolymphomas. Within lymphoma, the differential diagnosis includes several subtypes expressing PD-1 and other follicular T-cell markers, most notably primary cutaneous follicular helper T-cell lymphoma, MF, angioimmunoblastic T-cell lymphoma, and LyP. Additionally, PD-1 and CXCL13 expression is observed in primary cutaneous marginal zone lymphoma and primary cutaneous follicular center lymphoma [13,15].

Reported treatments for this condition vary significantly. The most commonly reported therapy is surgical excision, followed by radiotherapy. Other agents used include immunomodulators such as methotrexate, cyclophosphamide, and interferon-alpha-1b, as well as antibiotics (mainly doxycycline), topical corticosteroids, intralesional corticosteroids (as illustrated in our case), systemic corticosteroids, and phototherapy. The treatment outcomes described in the literature are consistent with the benign nature and favorable behavior of PCSM-TCLPD. A very high complete response rate (91.2–97.2%) and a very low recurrence rate (7.1%) have been reported. No mortality has been documented [12].

It has been suggested that the most common cutaneous reactions to SARS-CoV-2 vaccines may develop due to T-cell-mediated immune activation responses to vaccine components, including mRNA. Hooper et al. hypothesize that cutaneous lymphoid infiltrates are associated with immune system stimulation. This is supported by the review of the available literature, where no cases of primary cutaneous B-cell lymphoma have been reported. The recurrence of the disease may be attributed to the overproduction and exhaustion of CD4+ and CD8+ lymphocytes expressing CD30 after being stimulated by the vaccine [5]. Nevertheless, the activation of T lymphoid cells by mRNA vaccines appears to be in contrast to previous publications on cutaneous lymphoid hyperplasia associated with other vaccines. Such studies have suggested that the presence of foreign materials, such as vaccine adjuvants and viral proteins, can induce local B-cell hyperplasia in non-mRNA vaccines [16]. It is noteworthy that Moderna and Pfizer/BioNTech have developed different solutions to the challenge of RNA instability inherent in mRNA vaccine technology. This fact may explain the differences observed in this literature review between the adverse event profiles of the two formulations, especially in this issue, where the difference is even more apparent. When analyzing these data, it is also important to consider that BNT162b2 was the first vaccine available and the most widely used in different settings, which may have contributed to the increased reports of cutaneous adverse events [9,17].

Focusing on the PCSM-TCLPD cases, the two previously described patients had a typical clinical presentation with solitary lesions, indolent behavior, and a good response to treatment (Table 2). In contrast, our patient presented a less commonly described clinical presentation with a multifocal distribution (16 lesions), located in less common sites (mainly upper limbs and back), and with a younger age of onset. All cases occurred after BNT162b2 vaccination (two after the first dose and one after the third dose). The average time from vaccination to clinical onset was 10 days. None of the cases relapsed during the described follow-up.

## 5. Conclusions

To our knowledge, we present the third case of PCSM-TCLPD after SARS-CoV-2 vaccination and, notably, the first case with an atypical clinical presentation. Our case, along with a review of the literature, supports the etiologic link between COVID-19 immunization and T-cell cutaneous exacerbation. It is important to emphasize that these findings should not discourage vaccination. Instead, they underscore the importance of observation, comprehensive research, and extensive follow-up studies to elucidate the specific characteristics and underlying mechanisms of such manifestations.

## Figures and Tables

**Table 1:** Cutaneous lymphomas and cutaneous lymphoproliferative disorders reported after COVID-19 immunization.

Reference	Sex	Age (Years)	Lymphoproliferative Process	Vaccine	Time from the Dose of the Vaccine until the Onset	New Onset/Relapse
Brumfiel et al., 2021 [6]	Male	79	PCCD30-LPD	BNT162b2	2 days	Relapse
Panou et al., 2022 [7]	Male	60	CD30LCT-MF	ChAdOx1 nCoV-19	28 days	Relapse
Panou et al., 2022 [7]	Female	73	LyP type-A	ChAdOx1 nCoV-19	10 days	Relapse
Koumaki et al., 2022 [8]	Male	60	LyP type-D	ChAdOx1 nCoV-19	7 days	New onset
Koumaki et al., 2022 [8]	Female	66	LyP type-D	BNT162b2	10 days	New onset
Hooper et al., 2022 [9]	Female	70	CLH	BNT162b2	22 days	New onset
Hooper et al., 2022 [9]	Male	50	CLH	BNT162b2	28 days	New onset
Hooper et al., 2022 [9]	Male	50	LyP type-A	BNT162b2	4 days	New onset
Hooper et al., 2022 [9]	Female	20	LyP type-A	BNT162b2	42 days	New onset
Mintoff et al., 2021 [10]	Female	68	CLH	BNT162b2	7 days	New onset
Avallone et al., 2022 [5]	Female	47	CLH	BNT162b2	10 days	Relapse
Avallone et al., 2022 [5]	Male	67	LyP type-A	BNT162b2	15 days	Relapse
Avallone et al., 2022 [5]	Male	49	PCSM-TCLPD	BNT162b2	15 days	Relapse
Avallone et al., 2022 [5]	Male	58	SS	BNT162b2	4 days	Relapse
Avallone et al., 2022 [5]	Male	61	Erythrodermic MF	BNT162b2	14 days	Relapse
Avallone et al., 2022 [5]	Male	61	SS	BNT162b2	15 days	Relapse
Avallone et al., 2022 [5]	Male	55	CLH	BNT162b2	30 days	New onset
Avallone et al., 2022 [5]	Male	55	CLH	BNT162b2	7 days	New onset
Avallone et al., 2022 [5]	Female	80	SS	BNT162b2	15 days	New onset
Avallone et al., 2022 [5]	Male	60	LyP type-A	BNT162b2	30 days	New onset
Avallone et al., 2022 [5]	Female	52	PCSM-TCLPD	BNT162b2	3 days	New onset
Avallone et al., 2022 [5]	Female	61	LyP type-A	BNT162b2	10 days	New onset
Avallone et al., 2022 [5]	Male	45	PCSM-TCLPD	BNT162b2	20 days	New onset
Hobayan et al., 2023 [11]	Male	79	PCGDTCL	mRNA-1273	3 days	New onset

PCCD30-LPD: Primary cutaneous CD30-positive lymphoproliferative disorder. CD30LCT-MF: CD30 large cell transformation mycosis fungoides. LyP: Lymphomatoid papulosis. CLH: Cutaneous lymphoid hyperplasia. PCSM-TCLPD: Primary cutaneous CD4 small/medium T-cell lymphoproliferative disorder. SS: Sézary Syndrome. PCGDTCL: Primary cutaneous gamma/delta T-cell lymphoma.

**Table 2:** Primary cutaneous CD4 small/medium T-cell lymphoproliferative disorder reported after COVID-19 immunization.

Reference	Sex	Age	Vaccine	Onset (Days)	Localization	Number of Lesions	Therapy	Outcome	Follow-Up
Avallone et al., 2022 [5]	Female	52	BNT162b2 (First dose)	3	Forehead (nodule)	1	RT	CR	15 months
Avallone et al., 2022 [5]	Male	45	BNT162b2 (Third dose)	20	Left cheek (nodule)	1	SE	CR	3 months
Present case	Male	30	BNT162b2 (First dose)	7	Upper extremities and back (papulonodules)	16	Intralesional Triamcinolone	CR	14 months

RT: radiotherapy. CR: complete remission. SE: surgical excision.

## Data Availability

Data is contained within the article.
